# Cognitive Function Characteristics of Parkinson's Disease with Sleep Disorders

**DOI:** 10.1155/2017/4267353

**Published:** 2017-04-18

**Authors:** Jing Huang, Wenyan Zhuo, Yuhu Zhang, Hongchun Sun, Huan Chen, Peipei Zhu, Xiaobo Pan, Jianhao Yang, Lijuan Wang

**Affiliations:** ^1^Department of Neurology, Zhuhai People's Hospital, Zhuhai 519000, China; ^2^Department of Neurology, Guangdong Neuroscience Institute, Guangdong General Hospital and Guangdong Academy of Medical Sciences, Guangzhou 510080, China

## Abstract

*Objective*. The aim of this study was to investigate the cognitive function characteristics of Parkinson's disease (PD) with sleep disorders.* Methods*. Consecutive patients with PD (*n* = 96), patients with primary sleep disorders (*n* = 76), and healthy control subjects (*n* = 66) were assessed. The patients with PD were classified into sleep disorder (PD-SD) and non-sleep disorder (PD-NSD) groups.* Results*. Among 96 patients with PD, 69 were diagnosed with a sleep disorder. There were 38 sleep disorder cases, 31 RBD cases, and 27 NSD cases. On the Mini-Mental State Examination (MMSE), Montreal Cognitive Assessment (MoCA), and MoCA subtests, patients in the PD-SD, primary sleep disorder, and PD-NSD groups exhibited lower scores than those in the control group. Moreover, the PD-SD patients exhibited more significant cognitive impairment than was observed in the primary sleep disorder patients. In the PD-SD subgroup, the attention scores on the MoCA and on MoCA subtests were lower in the PD with RBD group than in the PD with insomnia group.* Conclusion*. PD with sleep disorders may exacerbate cognitive dysfunction in patients. PD associated with different types of sleep disorders differentially affects cognitive functions, and patients with PD with RBD exhibited poorer cognitive function than was seen in patients with PD with insomnia.

## 1. Introduction

Parkinson's disease (PD) is a typical movement disorder. In addition to motor symptoms, it is characterized by its nonmotor symptoms, including sleep disorders, cognitive dysfunction, autonomic dysfunction, and mental disorders, which seriously affect the quality of life of PD patients [1]. The most common nonmotor symptoms include sleep disorders and cognitive dysfunction. Sleep disorders affect up to 98% of PD patients [2]. At the time of PD diagnosis, 30–50% of patients may have mild cognitive impairment (MCI), but they usually do not show dementia unless the patient is diagnosed in the advanced stage of PD [3], and 60–80% of PD cases develop complete dementia within 10 years [4, 5]. Sleep quality is strongly correlated with health-related quality of life [6]. Sleep disorders include insomnia, sleep fragmentation, rapid eye movement sleep behavior disorder (RBD), sleep apnea syndrome, restless legs syndrome, and nocturnal enuresis [7]. In PD patients, insomnia and RBD, particularly the latter, are the most common and widely studied sleep disorders. RBD is not only a sleep disorder but also the preclinical manifestation of several neurodegenerative diseases (such as PD, multiple system atrophy, and Lewy body dementia) [8]. RBD includes a series of clinical symptoms (e.g., screaming, cursing and waving limbs, kicking, and falling out of bed). It accelerates the disease progression and affects the quality of sleep in PD patients, and it may lead to harm or death to patients or bedfellows [9].

The Braak pathological grade hypothesis drew attention to the fact that sleep disorders appear prior to cognitive impairment; moreover, there is a mutual influence between sleep disorders and cognitive impairment. It has been shown that chronic insomnia is an independent risk factor for cognitive impairment in nondepressed older men, whereas occasional insomnia does not increase the risk of cognitive impairment [10]. Similar to other neurodegenerative diseases (e.g., Alzheimer's disease), significant sleep problems occur in PD in combination with significant cognitive dysfunction [11]. Postuma et al. performed a 4-year follow-up study of nondementia PD patients. In their study, 27 cases of PD were associated with RBD, and the incidence of dementia in PD patients with RBD was 15% after 2 years, 29% after 3 years, and 48% after 4 years. In contrast, none of the 15 PD patients who did not present with RBD developed dementia [12]. Sleep disorders may represent an independent risk factor for cognitive impairment; however, most recent studies have only focused on sleep or cognitive conditions for PD patients. The relationship between cognitive function and PD with sleep disorders remains unclear, as do the effects of different types of sleep disorders on cognitive function.

Therefore, this study focused on PD with sleep disorders and comprehensively assessed the sleep quality and cognitive function in PD patients. We classified PD patients according to their sleep disorder types and analyzed the cognitive function characteristics of each subgroup to better characterize the relationship between PD with sleep disorders and cognitive function. Our study provides a novel theoretical basis and clinical application for the treatment, prevention, and early intervention of cognitive impairment in PD patients.

## 2. Materials and Methods

### 2.1. Study Population

#### 2.1.1. General Information

PD patients admitted to the Neurology Department of Guangdong General Hospital and Zhuhai People's Hospital from April 2013 to May 2016 were enrolled in our study. We also assessed a primary sleep disorder group and a healthy control group with the same age, sex distribution, and education level as the PD patients. All subjects were examined by experienced neurologists; they met the United Kingdom PD Society Brain Bank criteria [13] and did not receive anti-PD medication before the evaluation of sleep and the cognition scale. Exclusion criteria were as follows: (1) excessive daytime sleepiness (EDS), sleep attacks, sleep apnea syndrome, restless legs syndrome, parasomnia, and other types of sleep disorders; (2) other serious or unstable physical conditions that affect sleep and cognitive function assessment; (3) pain disorders, drug or alcohol addicts with severe physical illness, or sleep problems being directly a result of physical or mental effects; (4) severe anxiety or depression history and other mental disorders; (5) active epilepsy; (6) history of acute cerebrovascular disease in the previous 3 months; (7) use of sedative or hypnotic drugs within 2 weeks prior to enrollment; (8) presence of aphasia, delirium, or consciousness disorders that affect sleep and cognitive function assessment.

#### 2.1.2. Population Classification

According to the diagnostic criteria of sleep disorders, we classified the PD patients into 2 subgroups, the PD with sleep disorders (PD-SD) group and the PD with no sleep disorders (PD-NSD) group. The PD-SD group included the PD with insomnia group and the PD with RBD group [14], whereas the primary sleep disorder group was divided into the primary insomnia group and the primary RBD group. All patients with primary insomnia met the American Psychiatric Association Mental Disorders Diagnostic and Statistical Manual IV diagnostic criteria for primary sleep disorders [15], whereas all primary RBD patients fulfilled the International Sleep Disorders Classification II diagnostic criteria for RBD [16].

### 2.2. Study Methods

#### 2.2.1. Survey Demographics

We administered demographic surveys to all PD patients and to the control group, thereby obtaining general information regarding age, sex, height, weight, and education level and potential associations with other diseases (e.g., recent infection, high blood pressure, diabetes, cancer, and surgical history), age of onset, duration, drug treatment, family history, and concomitant symptoms.

#### 2.2.2. Clinical Assessments

*(a) Motor Function Assessment.* The motor function was assessed using the Unified PD Rating Scale Part III (UPDRS-III), and the disease severity was assessed using the Hoehn-Yahr (H-Y) grade.

*(b) Daily Capacity Assessment.* Activities of Daily Living Scale (ADL) was used.

*(c) Anxiety and Depression Assessment.* The Hamilton Anxiety Scale (HAMA) and the Hamilton Depression Scale (HAMD) were used.

*(d) Sleep Assessment.* The PDSS-2, PSQI, and ESS scales were administered to assess the quality of sleep.

*(e) Cognitive Function Assessment.* The Mini-Mental State Examination [MMSE] and Montreal Cognitive Assessment [MoCA] were used.

### 2.3. Statistical Methods

SPSS 13.0 statistical software was used for the statistical analysis. Measurement data are presented as the mean ± standard deviation (±sd). The Kolmogorov-Smirnov test method was adopted for the normality test. For two samples that exhibited a normal distribution, the means were compared using the two independent samples' *t*-test. In contrast, the samples noncompliant with a normal distribution were tested using the Mann–Whitney *U* test. When multiple samples obeyed the normal distribution and multiple sample averages were compared, one-way ANOVA (analysis of variance) was implemented. Levene's test for equality of variance and Welch's *t*-test for unequal variance were also utilized. The homogeneity of variance was analyzed by the LSD multiple comparison method, and unequal variance was evaluated by Dunnett's T3 multiple comparisons. Comparisons of multiple samples that were not normally distributed were performed using the nonparametric Kruskal-Wallis *H* test with count data tested using a chi-square test. When *P* < 0.05, we considered the difference to be statistically significant.

## 3. Results

### 3.1. General Information

The 96 PD patients included 41 males and 55 females (mean age 58.91 ± 12.19 years); 76 patients were included in the primary sleep disorders group, including 43 males and 33 females (mean age 57.30 ± 13.36 years). The normal control group consisted of 66 subjects, including 36 males and 30 females (mean age 56.63 ± 9.84 years).

According to the diagnostic criteria for PD sleep disorders, the PD patients included a PD-SD group of 69 cases, which accounted for 72.7% of the total PD patients (38 cases in the PD with insomnia group, or 40.1% of the total PD patients; 31 cases in the PD with RBD group, or 31.8%). There were 27 cases in the PD-NSD group, accounting for 27.3% of the total PD patients. The PD-SD and PD-NSD groups showed significant differences in the PDSS-2-T and PSQI-T scores but no significant differences in the ESS scores (Table 1). There were no significant differences in the age, age of onset, duration, years of education, UPDRS-III, H-Y classification, HAMA score, HAMD score, or ADL score (*P* > 0.05) (Table 2).

### 3.2. Cognitive Function Analysis in PD with Sleep Disorders

(1) There were significant differences in cognitive function among the PD-SD, PD-NSD, primary sleep disorder, and normal control groups. For the PD-SD and primary sleep disorder groups, the MMSE scores were only 25.48 ± 3.82 and 27.15 ± 3.65, and the MoCA scores were only 19.00 ± 4.99 and 22.54 ± 5.75, respectively, when using multiple independent samples. Similarly, for the overall cognitive function (MMSE and MoCA) and MoCA subtest assessments, the PD-SD and primary sleep disorder groups showed significantly lower scores (Table 3). Furthermore, using one-way ANOVA with the LSD test for cases in which Levene's test showed homogeneity of variance and with Dunnett's T3 method for cases of nonhomogeneous variance, we found that the PD-SD group showed more significant impairment of cognitive function than did the primary sleep disorder group, based on measures such as the MMSE and MoCA scores and the Naming, Attention, and Orientation subitems in the MoCA (Table 4).

(2) Comparing the subtypes of PD-SD, the MoCA score was lower in the PD with RBD group than in the PD with insomnia group. Similarly, the attention score of the MoCA subtest was lower in the PD with RBD group than in the PD with insomnia group (Table 5).

## 4. Discussion

To date, the combination of PD with sleep disorders and cognitive function has been poorly investigated, particularly regarding the difference between the effects of different types of sleep disorders on cognitive function. Adequate sleep is an important requirement for good memory and efficient executive function. The cognitive changes that occur in early-to-moderate PD are primarily deficits in executive function and memory [17–19], which are the same domains as those affected in individuals with insomnia [20]. Studies have investigated the impact of PD with RBD on cognitive function, but how PD with insomnia affects cognitive function remains poorly understood. To address these gaps, our study compares cognitive functions between PD patients with RBD and PD patients with insomnia. Gagnon et al. determined that the MCI incidence rate was 73% in PD patients with RBD but only 50% in primary RBD patients. Moreover, in the PD without RBD group, 11% of patients developed MCI, compared with an 8% incidence rate in the normal control group [21]. Furthermore, studies have indicated that there was no significant difference in motor function between PD with and without RBD. However, PD patients with RBD performed worse in memory, visuospatial, and executive functions [22]. Postuma et al. determined that PD patients with RBD were more likely to develop dementia than were PD patients without RBD [12]. Sleep quality in PD is significantly correlated with cognition, and it differentially impacts attention and executive function, thereby furthering our understanding of the link between sleep and cognition [23]. Our results were consistent with previous studies, and there were no significant differences in motor function or daily living ability between the PD with sleep disorder and PD with normal sleep groups. Interestingly, both PD with sleep disorder and primary sleep disorder patients showed significant impairments in the overall cognitive function and MoCA subtests compared with the PD with normal sleep and normal control groups. Moreover, the scores of the PD patients with sleep disorders were significantly lower than those of the primary sleep disorder patients. Thus, patients with PD with sleep disorders have a greater risk of cognitive dysfunction. Clinicians need to solve PD patients' sleep problems as early as possible in order to delay the associated cognitive decline.

In the analysis of the subtypes of PD with sleep disorders, the total MoCA score was lower in the PD with RBD group than in the PD with insomnia group. Furthermore, for the MoCA subtests, the PD patients with RBD had lower attention scores and more significant cognitive impairment compared with the PD patients with insomnia, particularly regarding attention. Therefore, poor sleepers exhibited worse performance on tests of global cognition. A sleep disorder is considered as an independent risk factor for PD-related cognitive impairment, whereas the impact of RBD on cognitive function is more obvious.

Cognitive function in patients with primary RBD has received increasing attention. RBD may be associated with preclinical presentation of a number of neurodegenerative diseases (multiple system atrophy, Lewy body dementia, and Parkinson's disease). The cognitive function of PD with RBD is also currently receiving substantial attention in the study of Parkinson's disease. Primary RBD patients often present with visuospatial impairment, attention dysfunction, executive dysfunction, or verbal memory loss [24]. Studies have indicated that 73% of PD patients with RBD present with MCI [25]. As previously discussed, RBD is considered an independent risk factor for PD-associated cognitive impairment. Consistent with other research results, our study demonstrated that PD patients with RBD have poorer cognitive function. To date, the pathophysiology underlying the effects of RBD on cognitive function remains unclear. First, the pathological mechanism of RBD may be related to the abnormalities of the tegmentum and the ascending reticular activating system [26]. Second, Braak et al. have suggested that the pathological evolution of PD that involves the medulla oblongata and pontine tegmentum occurs via a two-phase pathological process, and these are common regions of RBD occurrence [27]. The damaged regions of the cerebral cortex in PD or RBD patients are believed to regulate neural activity, and nervous system conduction disorders may affect cognitive function. It has been demonstrated that the brain metabolism perfusion patterns differ between PD and PD cognitive impairment patients. In PD cognitive impairment patients, there have been reports of reduced perfusion in the frontal and parietal regions and increased perfusion in the brain stem and cerebellum [28]. This abnormal metabolic perfusion has also been identified in studies of primary RBD [29]. The clinical manifestations of PD are significantly heterogeneous and may be related to the position and degree of neuronal injury. Furthermore, the mechanisms of neurodegeneration may differ between PD patients with and without RBD. However, there are no well-established imaging studies to elucidate the neuropathological mechanisms in PD with RBD; thus, additional studies are required to further illustrate its pathogenesis.

In summary, sleep disorders may exacerbate the occurrence and development of PD. Neurologists should focus on sleep disorders in PD patients and be fully aware of different types of sleep disorders to achieve early diagnosis, early intervention, and early treatment. This knowledge is essential for improving patient quality of life, reducing the risk of cognitive impairment, and slowing the progression of cognitive decline.

## Figures and Tables

**Table 1:** Comparison of subjective sleep in PD-SD and PD-NSD.

	PD-SD (69)	PD-NSD (27)	*Z*	*P* value
PDSS-2-T	20.69 ± 10.54	10.00 ± 5.90	−4.130	0.000
ESS-T	6.21 ± 4.39	4.39 ± 3.65	−1.496	0.135
PSQI-T	9.08 ± 4.22	6.44 ± 3.88	−2.329	0.020

**Table 2:** Comparison between PD-NSD and PD with sleep disorders.

	PD-SD (69)	PD-NSD (27)	*t*/*Z*/*χ*^2^	*P* value
Age, y	61.27 ± 11.38	56.28 ± 13.81	1.496	0.139
Gender, M/F (%)	32/37	11/16	0.091	0.763
Education, y	10.27 ± 3.92	12.39 ± 3.79	−1.805	0.071
Age of onset, y	57.25 ± 11.24	53.06 ± 12.87	1.298	0.199
PD duration, y	3.52 ± 2.96	3.31 ± 4.48	−1.042	0.297
UPDRS-III	28.23 ± 10.10	24.50 ± 10.84	−2.017	0.067
Hoehn-Yahr scale	2.68 ± 4.11	1.81 ± 0.94	−1.868	0.062
HAMA	9.33 ± 6.77	7.11 ± 3.61	−0.846	0.397
HAMD	9.69 ± 7.33	9.39 ± 6.09	−0.253	0.800
ADL	20.23 ± 9.87	19.83 ± 8.42	−0.610	0.701

**Table 3:** Cognitive function characteristic analysis comparing the PD-SD, PD-NSD, primary sleep disorder, and normal control groups.

	PD-SD (69)	PD-NSD (27)	Primary sleep disorders (76)	Normal controls (66)	*χ* ^2^	*P* value
MMSE	25.48 ± 3.82	28.44 ± 1.79	27.15 ± 3.65	28.92 ± 1.50	25.512	0.000
MoCA	19.00 ± 4.99	25.50 ± 3.20	22.54 ± 5.75	27.00 ± 2.49	47.963	0.000
Visuospatial and execution	2.50 ± 1.38	3.72 ± 1.02	3.15 ± 1.71	4.50 ± 0.83	34.282	0.000
Naming	2.35 ± 0.67	2.78 ± 0.55	2.87 ± 0.54	3.00 ± 0.00	37.522	0.000
Attention	4.50 ± 1.35	5.39 ± 0.92	5.48 ± 0.91	5.75 ± 0.44	27.669	0.000
Language	2.25 ± 0.91	2.78 ± 0.55	2.54 ± 0.59	2.75 ± 0.53	9.312	0.025
Abstract	0.96 ± 0.82	1.39 ± 0.70	1.15 ± 0.76	1.54 ± 0.66	10.108	0.018
Delayed recall	1.38 ± 1.10	3.61 ± 1.24	1.91 ± 1.76	3.58 ± 1.28	41.662	0.000
Orientation	5.08 ± 1.11	5.83 ± 0.38	5.70 ± 0.89	5.92 ± 0.28	26.226	0.000

**Table 4:** Cognitive function characteristic analysis comparing the PD-SD, PD-NSD, primary sleep disorders, and normal control groups.

	A versus B	A versus C	A versus D	B versus C	B versus D	C versus D
MMSE	0.004	0.038	0.000	0.590	0.922	0.043
MoCA	0.000	0.001	0.000	0.196	0.414	0.000
Visuospatial and execution	0.012	0.044	0.000	0.890	0.066	0.000
Naming	0.021	0.000	0.000	0.910	0.383	0.403
Attention	0.042	0.000	0.000	0.998	0.715	0.791
Language	0.052	0.816	0.062	0.345	1.000	0.440
Abstract	0.261	0.843	0.014	0.812	0.972	0.163
Delayed recall	0.000	0.427	0.000	0.000	1.000	0.000
Orientation	0.005	0.000	0.000	1.000	0.965	0.860

A: PD-SD; B: PD-NSD; C: primary sleep disorders; D: normal control.

**Table 5:** Cognitive function characteristic analysis comparing two subtypes of PD-SD.

	PD with insomnia (38)	PD with RBD (31)	*Z*	*P* value
MMSE	26.11 ± 3.86	24.67 ± 3.71	−1.914	0.056
MoCA	20.07 ± 5.36	17.62 ± 4.20	−2.187	0.029
Visuospatial and execution	2.74 ± 1.32	2.19 ± 1.44	−1.668	0.095
Naming	2.41 ± 0.69	2.29 ± 0.64	−0.746	0.456
Attention	4.93 ± 1.27	3.95 ± 1.28	−2.686	0.007
Language	2.26 ± 0.94	2.24 ± 0.89	0.161	0.872
Abstract	1.11 ± 0.80	0.76 ± 0.83	−1.466	0.143
Delayed recall	1.41 ± 1.25	1.33 ± 0.91	−0.011	0.991
Orientation	5.19 ± 1.18	4.95 ± 1.02	−1.247	0.212
